# Global Research Trends in Dietary Polyphenols for Preventing Non‐Communicable Chronic Diseases: A Bibliometric Study

**DOI:** 10.1002/fsn3.71539

**Published:** 2026-03-02

**Authors:** Xinjiang Huang, Pengyuan Kang, Haiying Chen, Jie Li, Jie Liu

**Affiliations:** ^1^ Department of Public Health Dazhou Central Hospital Dazhou Sichuan China; ^2^ Department of Microbiology and Center of Infectious Disease, School of Basic Medical Sciences Peking University Health Science Center Beijing China; ^3^ Department of Clinical Research Center Dazhou Central Hospital Dazhou Sichuan China; ^4^ Department of General Surgery Dazhou Central Hospital Dazhou Sichuan China

**Keywords:** cardiometabolic diseases, chronic disease prevention, dietary polyphenols, gut microbiota, metabolic syndrome, non‐communicable chronic diseases, oxidative stress

## Abstract

Dietary polyphenols, a diverse group of plant‐derived bioactives, are widely investigated for their potential to prevent non‐communicable chronic diseases (NCDs), but the structure and evolution of this research field remain unclear. This bibliometric study analyzed 3280 publications on dietary polyphenols and NCDs retrieved from Web of Science Core Collection, PubMed/MEDLINE, and DOAJ (1998–2024) to characterize temporal growth, global contributions, thematic hotspots, and the underlying journal knowledge base. Annual output rose from 13 papers in 1998 to more than 160 per year since 2016, indicating a transition from early expansion to a mature yet active stage. Research is led by teams in the United States, China, Japan, and Western Europe, with increasing participation from Brazil, India, Iran, and other emerging economies, but limited representation from sub‐Saharan Africa. Network and keyword analyses show that the literature is dominated by mechanistic in vitro and animal studies on oxidative stress, inflammation, and signaling pathways, alongside disease‐focused themes in cancer and cardiometabolic prevention. Recent hotspots include metabolic syndrome, obesity, high‐fat diet models, gut microbiota, and polyphenol‐rich dietary patterns, whereas large, long‐term human intervention trials remain relatively scarce. This study underscores the need for integrative, human‐focused, and globally inclusive research programs that test realistic polyphenol‐rich diets on clinically relevant NCD outcomes, and provides a quantitative map to guide priority‐setting for future nutrition and chronic disease research.

## Graphical abstract

This graphical abstract summarizes a bibliometric study on dietary polyphenols for preventing non‐communicable chronic diseases. It shows publication growth from 1998 to 2024, highlights core themes (oxidative stress, inflammation) and hotspots (metabolic syndrome, gut microbiota), and outlines future priorities like long‐term human studies.

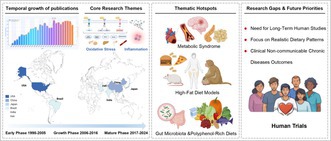

## 1 Introduction

Non‐communicable chronic diseases (NCDs) such as cardiovascular disease, type 2 diabetes, cancer, chronic kidney disease and neurodegenerative disorders now account for the majority of global morbidity and mortality, placing sustained pressure on health systems and economies worldwide (GBD 2021 Risk Factors Collaborators 2024; GBD 2023 Poland Collaborators 2025; Geng et al. 2025). Diet is a central, modifiable determinant of NCD risk, and current prevention strategies consistently emphasize patterns rich in plant‐derived foods and beverages rather than isolated nutrients. Within this context, dietary polyphenols—an abundant and structurally diverse family of bioactive compounds present in tea, coffee, cocoa, fruits, vegetables, whole grains, nuts, seeds and wine—have attracted considerable attention (Zekrumah et al. 2023; Rodriguez‐Mateos et al. 2025; Duarte et al. 2021; Cai et al. 2025). Over the past quarter‐century, experimental, epidemiological and clinical studies have explored their potential to modulate oxidative stress, low‐grade inflammation, vascular function, glucose and lipid metabolism, carcinogenesis and neurodegenerative pathways (Liu et al. 2025; Xie et al. 2023; Del Rio et al. 2013; Barik et al. 2025), raising the possibility that polyphenol‐rich diets could contribute meaningfully to the prevention and management of multiple NCDs across the life course.

Polyphenols encompass several major subclasses, such as flavonoids (e.g., flavonols, flavan‐3‐ols, anthocyanins), phenolic acids, stilbenes, and lignans, each comprising numerous individual compounds and conjugates. This chemical heterogeneity is mirrored by diversity in food sources, bioavailability profiles, metabolic pathways and biological targets (Manach et al. 2004; Quideau et al. 2011). Research in this field spans multiple levels of organization: in vitro models are used to characterize antioxidant, anti‐inflammatory, anti‐proliferative, and signaling effects; animal studies examine tissue‐level outcomes and interactions with the gut microbiota (Aryal et al. 2024; He et al. 2024); human intervention trials test specific foods, extracts, or supplements on intermediate risk markers; and observational cohorts assess long‐term associations between habitual intake and incident disease (Lanuza et al. 2023; Godos et al. 2024; Hadinata et al. 2025; Wang et al. 2025). At the same time, the interpretation of findings is complicated by variability in polyphenol content of foods, differences in analytical methods, inter‐individual variability in metabolism, and the fact that polyphenols are consumed as part of complex dietary patterns rather than in isolation (Raposo et al. 2024; Favari et al. 2024; Scavuzzi and Dichi 2025). As countries update food‐based dietary guidelines to reflect emerging evidence on plant‐rich diets and bioactive compounds (Tomaszewski 2023; Markscheffel and Schröter 2021; Klarin 2024), there is growing interest in understanding how knowledge about dietary polyphenols and NCD prevention has accumulated and how it is organized across disciplines, populations, and disease areas (de Freitas Marinho et al. 2024; Sheng et al. 2025; Harris et al. 2025).

Numerous narrative and systematic reviews, including meta‐analyses, have summarized the health effects of specific polyphenol classes or food sources on particular endpoints like cardiovascular disease, type 2 diabetes, obesity, site‐specific cancers, or cognitive decline (Wu et al. 2024; Chew et al. 2024; Ammar et al. 2020; Xu et al. 2021). Other overviews have focused on mechanistic aspects, including redox biology, endothelial function, lipid oxidation, insulin signaling and interactions with the intestinal microbiota. More recently, research has expanded to encompass polyphenol‐rich dietary patterns, omics‐based approaches and personalized nutrition (Ottaviani et al. 2023; Xia et al. 2023; Salomone et al. 2020). However, despite this prolific output, there has been limited systematic attention to the structure and evolution of the research field itself. Existing bibliometric analyses tend to be restricted to single compounds, single subclasses (e.g., flavonoids), specific diseases or limited time windows, and thus cannot fully capture how global activity on dietary polyphenols and NCDs has developed over the last two decades. Recent studies exemplify this continued focus on isolated endpoints rather than the field as a whole (Vázquez‐Ruiz et al. 2022; Laveriano‐Santos et al. 2025; Wu et al. 2025; Hu et al. 2025). This lack of an overarching, quantitative perspective makes it difficult to answer several important questions: How has research volume on dietary polyphenols and NCDs grown over time, and has the field reached a mature stage or is it still in a phase of rapid expansion? Which countries, institutions and collaborative networks have driven this growth, and how are contributions distributed between long‐standing and emerging research hubs? How have core thematic areas—such as antioxidant mechanisms, cardiovascular prevention, metabolic syndrome, cancer chemoprevention and microbiota‐related pathways—emerged, interacted and potentially shifted in prominence? And to what extent do current publication and citation patterns reveal imbalances between mechanistic work (Intended to elucidate the biological mechanism of action) and human studies, or between different NCD categories and polyphenol exposures?

Bibliometric methods offer a structured approach to address these questions by combining quantitative indicators with network and temporal analyses of large literature sets. In other biomedical fields, such approaches have delineated developmental stages, mapped collaboration, and identified research fronts (Amiruddin et al. 2025; Taha and Abdelwahab 2026). Applied here, bibliometric mapping can reveal not only collaboration networks but also how research foci—including disease targets, exposure modalities, and mechanistic concepts—cluster and evolve. This can help identify understudied areas, inform funding priorities, and support the translation of mechanistic insights into dietary guidance.

Therefore, this study aims to support more strategic, inclusive, and translationally relevant research agendas in nutrition and chronic disease prevention.

## 2 Methods

### 2.1 Data Sources and Search Strategy

This bibliometric study focused on peer‐reviewed literature addressing dietary polyphenols in relation to non‐communicable chronic diseases (NCDs). To capture both mechanistic and clinical strands of research, we combined three complementary databases: Web of Science Core Collection (Science Citation Index Expanded and Emerging Sources Citation Index), PubMed/MEDLINE, and the Directory of Open Access Journals (DOAJ). Searches covered publications dated from 1 January 1998 to 31 December 2024, and were completed on the same calendar day to avoid temporal bias in citation counts.

Search strategies were tailored to the indexing characteristics of each database but maintained a common conceptual structure. For the exposure component, controlled vocabulary (MeSH terms in PubMed) and free‐text variants were used for “polyphenols”, “phenolics”, “flavonoids”, “flavonols”, “flavan‐3‐ols”, “anthocyanins”, “proanthocyanidins”, “stilbenes”, “lignans”, “tannins”, and frequently studied individual compounds (e.g., resveratrol, quercetin, epigallocatechin gallate). To restrict the retrieval to nutrition‐relevant content, these terms were combined with food‐related expressions such as “diet*”, “food*”, “beverage*”, “tea”, “wine”, “fruit*”, “vegetable*”, “cereal*”, or “cocoa”.

For the outcome component, we included umbrella terms for NCDs and specific disease groups. Search strings combined “non‐communicable disease*”, “chronic disease*”, “lifestyle disease*”, “cardiovascular disease*”, “coronary heart disease”, “stroke”, “hypertension”, “atherosclerosis”, “diabetes mellitus”, “obesity”, “metabolic syndrome”, “dyslipidemia”, “cancer”, “tumor*”, “neoplasm*”, “neurodegenerative disease*”, “Alzheimer*”, “Parkinson*”, “chronic kidney disease”, and “chronic respiratory disease*”. Mechanistic proxies of NCD risk such as “insulin resistance”, “oxidative stress”, “inflammation”, and “endothelial dysfunction” were included when explicitly linked to long‐term cardiometabolic or oncologic outcomes.

The final Boolean structure in each database combined polyphenol‐related and disease‐related terms with “AND”, and individual synonyms with “OR”, restricted to title, abstract, and keyword fields where possible. In PubMed the search was implemented using a combination of MeSH headings and text words in Title/Abstract; in Web of Science the search was run in the “Topic” field; in DOAJ it was applied to all available metadata fields. Publication type filters were set to retain only “Article” and “Review”. Language was limited to English to ensure comparability of bibliographic fields and citation metrics across sources. All retrieved records were exported with full metadata and cited references in their native formats (MEDLINE, BibTeX, and plain text) and subsequently harmonized into Web‐of‐Science‐compatible plain text using the R‐bibliometrix conversion routines for downstream analysis in CiteSpace, VOSviewer, and R.

The following search terms were employed to retrieve literature from Doaj: (polyphenol* OR phenolic* OR flavonoid* OR flavonol* OR “flavan‐3‐ol*” OR anthocyanin* OR proanthocyanidin* OR stilbene* OR lignan* OR tannin* OR resveratrol OR quercetin OR “epigallocatechin gallate” OR EGCG) AND (diet* OR food* OR beverage* OR tea OR wine OR fruit* OR vegetable* OR cereal* OR cocoa) AND (“non‐communicable disease*” OR “chronic disease*” OR “lifestyle disease*” OR “cardiovascular disease*” OR “coronary heart disease” OR stroke OR hypertension OR atherosclerosis OR “diabetes mellitus” OR obesity OR “metabolic syndrome” OR dyslipidemi* OR cancer OR tumor* OR neoplasm* OR “neurodegenerative disease*” OR Alzheimer* OR Parkinson* OR “chronic kidney disease” OR “chronic respiratory disease*” OR “insulin resistance” OR “oxidative stress” OR inflammation OR “endothelial dysfunction”) AND bibjson.journal.language:en AND bibjson.year:[1998 TO 2024].

The following search terms were employed to retrieve literature from Web of Science: TS = ((polyphenol* OR phenolic* OR flavonoid* OR flavonol* OR “flavan‐3‐ol*” OR anthocyanin* OR proanthocyanidin* OR stilbene* OR lignan* OR tannin* OR resveratrol OR quercetin OR “epigallocatechin gallate” OR EGCG) AND (diet* OR food* OR beverage* OR tea OR wine OR fruit* OR vegetable* OR cereal* OR cocoa) AND (“non‐communicable disease*” OR noncommunicable disease* OR “chronic disease*” OR “lifestyle disease*” OR “cardiovascular disease*” OR “coronary heart disease” OR stroke OR hypertension OR atherosclerosis OR “diabetes mellitus” OR obesity OR “metabolic syndrome” OR dyslipidemi* OR cancer OR tumor* OR tumor* OR neoplasm* OR “neurodegenerative disease*” OR Alzheimer* OR Parkinson* OR “chronic kidney disease” OR “chronic respiratory disease*” OR “insulin resistance” OR “oxidative stress” OR inflammation OR “endothelial dysfunction”)) AND (PY=(1998‐2002) OR PY=(2003‐2007) OR PY=(2008‐2012) OR PY=(2013‐2017) OR PY=(2018‐2022) OR PY=(2023‐2024)).

The following search terms were employed to retrieve literature from Pubmed: ((“Polyphenols”[MeSH Terms] OR polyphenol*[Title/Abstract] OR phenolic*[Title/Abstract] OR flavonoid*[Title/Abstract] OR flavonol*[Title/Abstract] OR “flavan‐3‐ol*”[Title/Abstract] OR anthocyanin*[Title/Abstract] OR proanthocyanidin*[Title/Abstract] OR stilbene*[Title/Abstract] OR lignan*[Title/Abstract] OR tannin*[Title/Abstract] OR resveratrol[Title/Abstract] OR quercetin[Title/Abstract] OR “epigallocatechin gallate”[Title/Abstract] OR EGCG[Title/Abstract]) AND (diet*[Title/Abstract] OR food*[Title/Abstract] OR beverage*[Title/Abstract] OR tea[Title/Abstract] OR wine[Title/Abstract] OR fruit*[Title/Abstract] OR vegetable*[Title/Abstract] OR cereal*[Title/Abstract] OR cocoa[Title/Abstract]) AND (“Noncommunicable Diseases”[MeSH Terms] OR “Chronic Disease”[MeSH Terms] OR “Cardiovascular Diseases”[MeSH Terms] OR “Coronary Disease”[MeSH Terms] OR “Stroke”[MeSH Terms] OR “Hypertension”[MeSH Terms] OR “Atherosclerosis”[MeSH Terms] OR “Diabetes Mellitus”[MeSH Terms] OR “Obesity”[MeSH Terms] OR “Metabolic Syndrome”[MeSH Terms] OR “Dyslipidemias”[MeSH Terms] OR “Neoplasms”[MeSH Terms] OR “Neurodegenerative Diseases”[MeSH Terms] OR “Alzheimer Disease”[MeSH Terms] OR “Parkinson Disease”[MeSH Terms] OR “Kidney Diseases, Chronic”[MeSH Terms] OR “Respiratory Tract Diseases”[MeSH Terms] OR “Insulin Resistance”[MeSH Terms] OR “Oxidative Stress”[MeSH Terms] OR inflammation[MeSH Terms] OR “endothelial dysfunction”[Title/Abstract])) AND (english[lang]) AND (“1998/01/01”[Date ‐ Publication] : “2024/12/31”[Date ‐ Publication]) AND (journal article[Publication Type] OR review[Publication Type]).

### 2.2 Study Selection and Eligibility Criteria

The initial search output from the three databases contained overlapping and heterogeneous records. Bibliographic files were first imported into R (version 4.x) and converted to a unified data frame using the convert2df function in the bibliometrix package. Automated de‐duplication relied on exact matching of Digital Object Identifiers (DOIs) and near‐exact matching of combinations of title, first author, publication year, and journal. Potential duplicates flagged by the algorithm were inspected manually, and when multiple records referred to the same article, the most complete entry was retained.

Eligibility criteria were defined a priori. Records were considered eligible if they met all of the following conditions: the publication was a full‐length original article or narrative/systematic review in a peer‐reviewed journal; at least one dietary polyphenol or polyphenol‐rich food, beverage, or extract was a central exposure; at least one NCD or its established chronic pathophysiological pathway (e.g., atherosclerosis, insulin resistance, carcinogenesis, neurodegeneration) was a central outcome; and the article clearly linked the polyphenol exposure to prevention, progression, prognosis, or mechanistic underpinnings of NCDs. Experimental studies using cell lines or animal models were eligible when their rationale or discussion explicitly addressed chronic disease contexts rather than acute toxicity or short‐term pharmacology.

We excluded conference abstracts, meeting proceedings, editorials, commentaries, letters, corrections, book chapters, and dissertations. Articles focused solely on analytical chemistry, food technology, or plant science without any link to human or animal health were removed. Similarly, studies examining synthetic phenolic drugs, non‐dietary industrial phenolics, or acute poisoning were excluded. Records without essential bibliographic information (missing title, year, or journal) that could not be reliably completed from cross‐checking were also omitted.

Two reviewers (Pengyuan Kang and Haiying Chen) independently screened titles and abstracts against these criteria, followed by full‐text inspection when relevance was uncertain. Disagreements were resolved by discussion with a third senior reviewer (Jie Liu) until consensus was reached. Screening was performed iteratively, with periodic calibration exercises to maintain a consistent interpretation of inclusion rules. At the end of this process, 3280 articles published between 1998 and 2024 were retained as the final dataset for bibliometric analysis, and their annual distribution was used to derive publication trends over time.

### 2.3 Data Extraction, Normalization, and Quality Control

For each included article we extracted all available bibliographic and citation fields supplied by the databases: title, abstract, author names, author affiliations, corresponding author, journal, year of publication, volume, issue, pages, Digital Object Identifier, author keywords, database‐assigned keywords (e.g., MeSH terms), country and institution information, document type, and total citation counts. When multiple databases supplied differing citation counts, Web of Science values were used as the primary reference because of their compatibility with the science‐mapping tools employed.

Substantial effort was devoted to harmonizing names of authors, institutions, and countries, as inconsistencies at this stage can distort network structures. Using custom R scripts in combination with bibliometrix utilities, we standardized country names (e.g., “USA”, “U.S.A.”, “United States of America” as “United States”), merged alternative spellings of institutions (e.g., “Harvard Univ”, “Harvard University”, “Harvard School of Public Health”), and unified author identities where initials or transliteration varied. Ambiguous high‐productivity authors were checked manually using journal websites, ORCID records, and cross‐reference of research topics to minimize erroneous splitting or merging of identities. Affiliations were parsed to assign each article to one or more countries on the basis of institutional addresses, enabling subsequent classification into single‐country and international collaborations.

Keyword processing combined automated text‐mining and manual curation. All author keywords and database keywords were converted to lower case, stripped of punctuation, and lemmatized to merge singular and plural forms. A domain‐specific thesaurus was constructed to group synonymous or closely related terms for polyphenol classes (e.g., “flavan‐3‐ols” and “catechins”), food sources (“green tea” and “ *Camellia sinensis* ”), and disease entities (“type 2 diabetes mellitus” and “T2DM”). Non‐informative words (e.g., “study”, “effect”, “model”) and generic epidemiological terms (e.g., “risk factor”, “cohort”) were removed. The cleaned keyword list was used to generate both co‐occurrence networks and time‐trend analyses.

Records labeled as retracted, withdrawn, or expression of concern in any database were identified and removed. A random 5% sample of records was re‐checked manually to verify correct normalization of authors, institutions, and keywords.

### 2.4 Performance Indicators and Collaboration Network Analyses

To describe the quantitative development of research on dietary polyphenols and NCDs, we first conducted a performance analysis using R‐bibliometrix. Annual numbers of publications from 1998 to 2024 were calculated, along with cumulative counts and yearly growth rates. The temporal trajectory was assessed against Price's law by fitting exponential and logistic models to the annual output series in R using non‐linear least squares. This allowed delineation of formative, exponential, and potential saturation phases of the field.

Productivity and impact at the level of countries, institutions, journals, and authors were evaluated using standard bibliometric indicators: total publications, total citations, average citations per article, and h‐index. For countries, we applied fractional counting so that each multi‐country article contributed 1/n to each participating country, whereas descriptive analyses at the article level used full counting. International collaboration patterns were characterized by classifying articles into four collaboration classes (single‐author, single‐country, multi‐institution, and multi‐country) based on the affiliation metadata. The proportion of internationally co‐authored articles over time was used as a proxy for globalization of the field.

Co‐authorship networks were constructed at country, institutional, and author levels using VOSviewer (version 1.6.19). For each level, a minimum productivity threshold was imposed to maintain interpretability of the maps (e.g., at least five documents per country, 10 per institution, and eight per author). Edges between nodes represented the number of shared publications, and association‐strength normalization was applied to account for differences in node size. Network layouts were generated with the default VOS mapping algorithm; nodes were color‐coded by modularity‐based clusters that approximate collaborative communities. Centrality measures, including degree and betweenness, were calculated to identify hubs and bridging entities in the collaboration structure.

CiteSpace (version 6.x) was used to complement these analyses with time‐sliced collaboration networks, enabling visualization of how key countries and institutions entered the field and how their roles evolved. Time slicing was performed in one‐year intervals from 1998 to 2024, with the top‐N per slice selection strategy and pathfinder pruning to simplify network topology. Together, these procedures yielded a coherent picture of global and national collaboration in polyphenol‐NCD research, including the balance between domestic and international partnerships and the emergence of influential research clusters.

### 2.5 Thematic Mapping and Temporal Evolution of Research Topics

Scientific themes and their evolution were examined primarily through keyword‐based analyses. After cleaning and normalization, all author keywords and database‐assigned keywords were imported into VOSviewer to generate co‐occurrence networks. A minimum occurrence threshold (typically 10 occurrences) was set to focus on stable and influential terms. The resulting network was clustered using VOSviewer's modularity optimization, producing groups of related keywords corresponding to thematic areas such as cardiovascular prevention, glycemic control, cancer chemoprevention, gut microbiota, or molecular mechanisms like oxidative stress and inflammation. Network visualizations were generated in the standard layout and in density mode, where the color gradient reflects local concentration of high‐frequency terms.

To explore the dynamics of topic emergence and decline, overlay visualizations in VOSviewer were used, in which the average publication year of each keyword is mapped onto a color scale. This highlights early‐stage topics and recent hotspots. Complementarily, the trendTopics and thematicEvolution functions in R‐bibliometrix were applied to track the temporal trajectories of the most frequent keywords and to examine how thematic clusters reconfigured across pre‐specified time periods (1998–2007, 2008–2015, 2016–2024). These routines rely on correspondence analysis and Sankey‐style visualizations to reveal whether, for example, early antioxidant‐focused research converged into more recent microbiome, epigenetics, or omics‐based themes.

CiteSpace was employed for more granular time‐slice analyses. Using the same 1998–2024 window and one‐year slices, we constructed keyword co‐occurrence networks in each slice with the g‐index (*k* = 25) as the selection criterion. The software's “timeline” and “timezone” views were used to visualize the persistence and succession of thematic clusters across years. To identify abrupt increases in attention, we applied Kleinberg's burst‐detection algorithm to keywords, with a minimum burst duration of 2 years and default *γ* parameters. This yielded a ranked list of keywords showing the strongest citation or occurrence bursts, which are interpreted as markers of emerging fronts, such as shifts from isolated compound studies toward polyphenol‐rich dietary patterns, or from in vitro antioxidant assays toward clinical outcomes and gut microbiota interactions.

Descriptive term‐frequency analyses were carried out in R to compute absolute and relative frequencies of all cleaned keywords. These were visualized using bubble plots and word clouds to provide an intuitive overview of dominating concepts while retaining the quantitative information needed for formal mapping. Together, these procedures connected static network structures with temporal information, enabling a nuanced reconstruction of how research on dietary polyphenols and NCDs has unfolded over the last quarter‐century.

### 2.6 Source Journal and Knowledge‐Base Analyses

To characterize the journals that constitute the publication venues and intellectual base of polyphenol–NCD research, we undertook both source and cited‐journal analyses. For each source journal in which at least one included article appeared, bibliometric indicators were computed using R‐bibliometrix: total publications, total citations, average citations per article, local citation score within the dataset, and h‐index derived from local citations. Journals were ranked by these indicators, and Bradford's law was applied to identify the core set of journals accounting for the largest share of articles in the field. Temporal trends in journal productivity were assessed to explore whether newer nutrition, food science, or clinical journals were gaining prominence.

Journal‐level co‐occurrence and co‐citation networks were generated in VOSviewer. In the co‐occurrence network, nodes represented source journals linked by cross‐citation relations derived from the reference lists of the 3280 articles. A minimum threshold for the number of published articles was imposed to restrict the network to influential journals, and association‐strength normalization was again used. Clustering revealed groups of journals corresponding to broad disciplinary domains, such as nutrition and dietetics, cardiometabolic medicine, oncology, and basic biochemistry or molecular biology.

CiteSpace was used to map the knowledge base through cited‐journal co‐citation analysis. Using the same yearly time slices, the most frequently co‐cited journals in each slice were selected with the g‐index criterion, and co‐citation networks were constructed and pruned using pathfinder and pruning sliced networks. Citation‐burst detection at the journal level identified titles whose influence on the field increased sharply over limited periods, providing insight into shifts in preferred publication outlets or methodological standards.

In addition, a dual‐map overlay of journals was produced in CiteSpace; citation trajectories linking the two sides highlight the principal flows of knowledge, for example, from nutrition and internal‐medicine journals to foundational journals in chemistry, molecular biology, or epidemiology. These combined analyses of source and cited journals delineated both the visible publication landscape and the deeper knowledge structures underpinning research on dietary polyphenols in the prevention and management of non‐communicable chronic diseases.

## 3 Results

### 3.1 Overview of the Retrieved Literature and Temporal Trends

**FIGURE 1:**
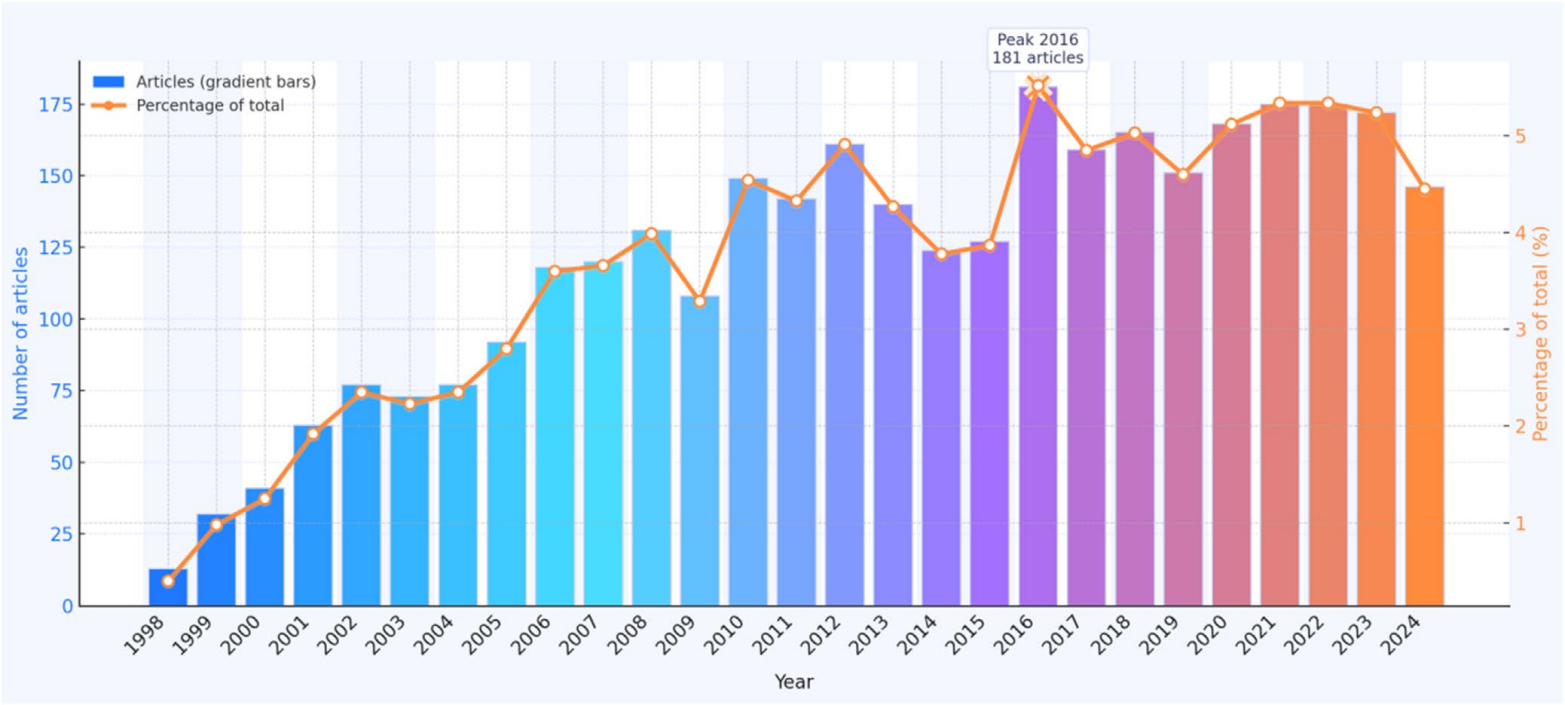
Annual number and proportion of publications on dietary polyphenols and non‐communicable chronic diseases, 1998–2024.

A total of 3280 eligible articles on dietary polyphenols in relation to non‐communicable chronic diseases were identified between 1998 and 2024. The annual distribution of these publications and their proportion of the entire dataset are summarized in Table 1 and visualized in Figure 1. The first indexed articles appeared in 1998, with only 13 publications (0.40% of the total). From 1999 onwards, the field expanded steadily: yearly output rose from 32 articles in 1999 to 92 in 2005, corresponding to an early growth phase in which the annual number of papers more than doubled approximately every 5 years. Over this initial period (1998–2005), 468 articles were published, accounting for 14.3% of all documents.

**TABLE 1:** Annual publication volume.

Year	Articles	Percentage of the total
1998	13	0.40
1999	32	0.98
2000	41	1.25
2001	63	1.92
2002	77	2.35
2003	73	2.23
2004	77	2.35
2005	92	2.80
2006	118	3.60
2007	120	3.66
2008	131	3.99
2009	108	3.29
2010	149	4.54
2011	142	4.33
2012	161	4.91
2013	140	4.27
2014	124	3.78
2015	127	3.87
2016	181	5.52
2017	159	4.85
2018	165	5.03
2019	151	4.60
2020	168	5.12
2021	175	5.34
2022	175	5.34
2023	172	5.24
2024	146	4.45

From 2006 to 2015, the literature entered a consolidation phase characterized by sustained expansion and increasing diversification of topics. As shown in Table 1, annual publications exceeded 100 from 2006 onwards and fluctuated between 108 and 161 articles per year. Compared with 1998, the output in 2012 was more than twelvefold higher, and the cumulative number of papers in this decade (1320 articles) represented 40.2% of the total corpus. Although minor fluctuations were observed around 2009 and 2013, the overall trajectory remained upward, indicating that research on polyphenols and chronic disease prevention had become an established domain rather than a nascent niche.

The most recent period (2016–2024) is marked by high but stable productivity. The annual number of articles peaked in 2016 with 181 publications, representing 5.52% of all retrieved documents. Thereafter, output remained consistently high, ranging from 146 to 175 articles per year, with an average of approximately 166 articles. This period contributed nearly half of all publications (1492 articles; 45.5% of the dataset), indicating that research activity in this area has plateaued at a mature and sustained level rather than declining. The modest decrease in 2024 likely reflects incomplete indexing of the most recent year rather than a genuine downturn in research interest.

**FIGURE 2:**
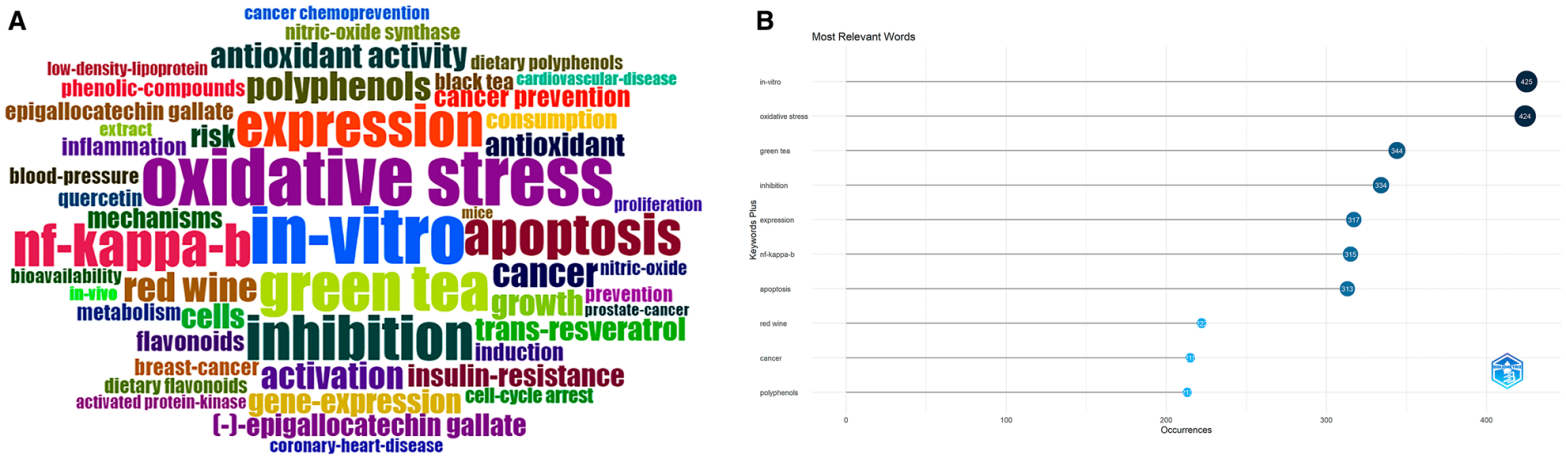
Distribution of high‐frequency themes in dietary polyphenol and NCD research. (A) Most frequent keywords plus in the dietary polyphenol–NCD literature. (B) Word cloud of high‐frequency terms related to dietary polyphenols and non‐communicable chronic diseases.

To provide an initial qualitative impression of the thematic orientation of the corpus, we examined the most frequent Keywords Plus terms. As shown in Figure 2A, the leading terms were “in vitro” (425 occurrences) and “oxidative stress” (424 occurrences), followed by “green tea” (344), “inhibition” (334), “expression” (317), “NF‐kappa‐B” (315), and “apoptosis” (313). Additional high‐frequency terms, such as “red wine”, “cancer”, and “polyphenols”, also occurred more than 200 times, underscoring the centrality of experimental models, oxidative and inflammatory pathways, and cancer‐related outcomes in this literature. The word cloud constructed from all high‐frequency terms further highlights these patterns (Figure 2B). Large word clusters related to “oxidative stress”, “in vitro”, “green tea”, “inhibition”, “apoptosis”, “insulin‐resistance”, “coronary‐heart‐disease”, “epigallocatechin gallate”, “quercetin”, and “trans‐resveratrol” indicate that investigations have predominantly focused on mechanistic links between specific polyphenol‐rich foods or compounds and key biological processes relevant to cardiometabolic and oncologic diseases. Together, the temporal and lexical overviews show that research on dietary polyphenols and NCDs has expanded rapidly since the late 1990s and remains an active, mechanistically oriented field of inquiry.

### 3.2 Geographic Distribution of Research Output and International Collaboration

**FIGURE 3:**
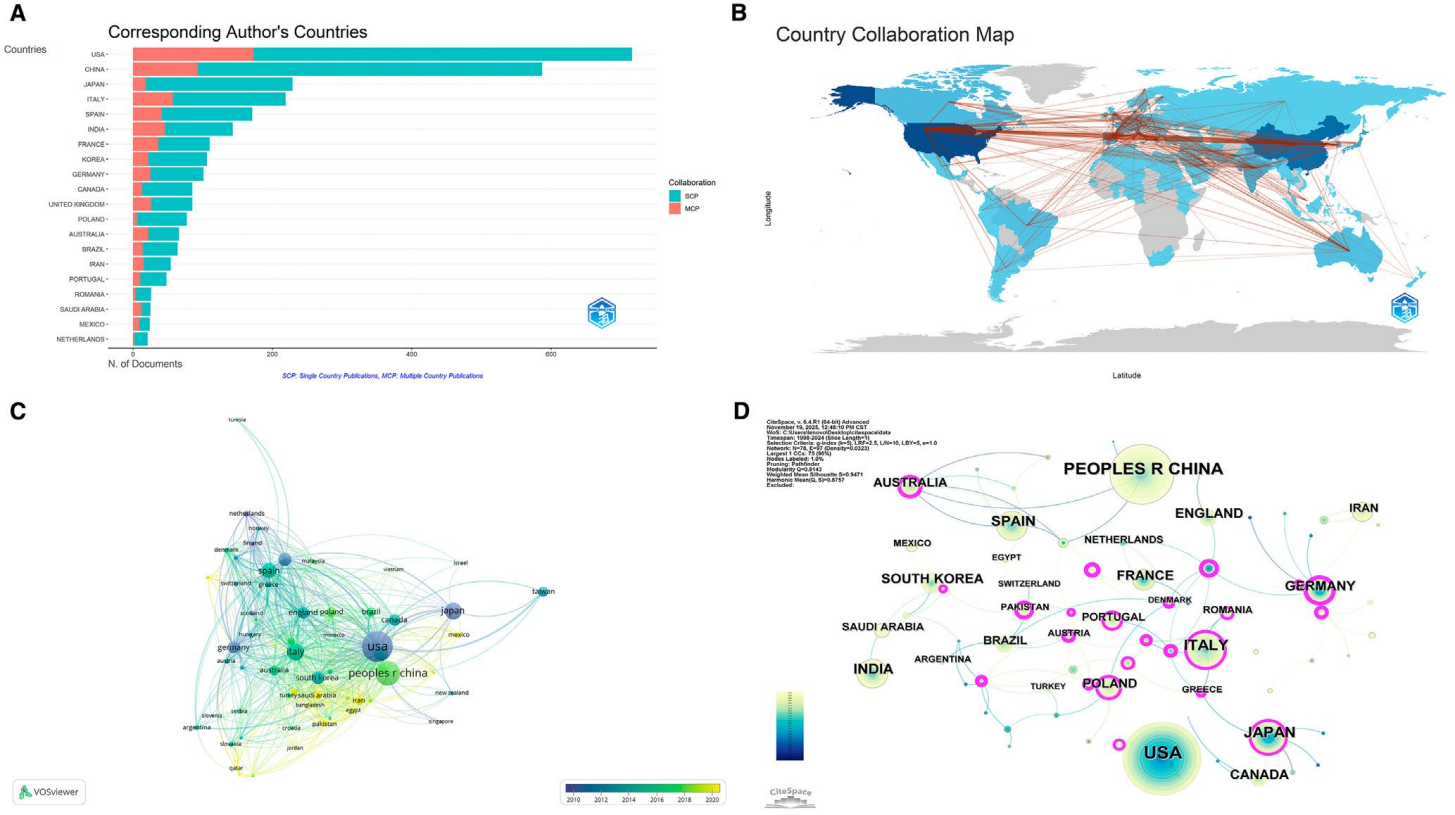
Global research output distribution and international collaboration networks. (A) Distribution of articles by corresponding author's country and type of collaboration (SCP vs. MCP). (B) Global map of international country collaborations in dietary polyphenol–NCD research. (C) Country co‐authorship network based on VOSviewer overlay visualization. (D) Country collaboration network with betweenness centrality derived from CiteSpace.

The 3280 articles included in this study originated from a broad range of countries and regions, indicating that research on dietary polyphenols and non‐communicable chronic diseases has become a genuinely global enterprise. The distribution of publications according to the corresponding author's country is summarized in Table 2 and depicted in Figure 3A. As shown in Figure 3A, the United States occupies the leading position in terms of number of papers, followed by China and Japan. A second tier of high‐productivity countries includes several European nations (Italy, Spain, France, Germany, the United Kingdom, and Poland), as well as India, South Korea, Canada, Australia, and Brazil. Several emerging economies in the Middle East and Latin America (e.g., Iran, Saudi Arabia, Mexico, and Romania) contribute a smaller but non‐negligible share of the literature, indicating increasing participation from outside the traditional research hubs.

**TABLE 2:** Major contributing countries and their collaboration profiles in research on dietary polyphenols and non‐communicable chronic diseases.

Collaboration profile	Qualitative description of output and collaboration pattern	Representative countries
High‐output, globally connected hubs	Very high volume of publications; extensive participation in multi‐country publications; act as central nodes linking Europe, North America, and Asia–Pacific in the collaboration networks	United States, China, Japan, Italy, Spain, France, Germany, United Kingdom, Canada, Australia
High‐output, predominantly domestic collaborations	Substantial publication output with a larger share of single‐country publications; international links present but less dense than in global hubs	India, Poland, Brazil, Iran, South Korea
Medium‐output, regionally integrated partners	Moderate number of publications; active collaboration within regional clusters (e.g., intra‐European or Asia–Pacific) and selective links to global hubs	Portugal, Netherlands, Switzerland, Austria, Greece, Mexico, Argentina
Emerging contributors with growing international ties	Smaller but increasing output; collaboration lines mainly directed toward one or more global hubs, indicating progressive integration into the international research network	Saudi Arabia, Romania, Turkey, Qatar, Pakistan, Bangladesh, Egypt, Tunisia

Figure 3A also distinguishes publications produced within a single country (SCP) from those arising from multi‐country collaborations (MCP). The leading producers, such as the United States and China, show a substantial absolute volume of SCP articles, reflecting strong domestic research capacity. At the same time, MCP papers constitute an important fraction of their output, particularly for countries such as the United Kingdom, Canada, Australia, and several European nations, underscoring the importance of cross‐border collaboration for high‐impact work. In contrast, some emerging contributors display a more nationally focused pattern, with a higher proportion of SCP manuscripts, suggesting that international partnerships in these settings are still developing. Table 2 qualitatively groups countries into collaboration profiles—high‐output and highly internationalized, high‐output but predominantly domestic, and moderate‐output emerging contributors—providing a concise overview of the global landscape. The spatial configuration of these collaborations is illustrated by the world map of country co‐authorship (Figure 3B). As shown in Figure 3B, dense linkages connect North America, Western Europe, and East Asia, forming a tri‐polar core of collaborative activity. The United States acts as a major hub linking Europe with Asia and Oceania, whereas intra‐European collaboration forms a tightly interwoven subnetwork. Numerous, though generally thinner, links extend from this core to South America, the Middle East, and parts of Africa, highlighting the gradual diffusion of expertise and shared projects beyond the most research‐intensive regions. Network analyses further clarify the roles of individual countries. The VOSviewer‐based co‐authorship network (Figure 3C) reveals that the United States and China occupy central positions with large node sizes and multiple connections, closely linked to Japan, Italy, Spain, Germany, France, and the United Kingdom. The color gradient in Figure 3C suggests that several Asian and Middle Eastern countries joined the network more recently, with more recent average publication years than long‐standing participants such as the United States and Western European countries. Distinct clusters correspond broadly to European, East Asian, and trans‐Pacific cooperation groups, yet these clusters are highly interconnected, emphasizing the integrative nature of the field. Complementary insights are provided by the CiteSpace country collaboration map (Figure 3D). In this representation, node size reflects overall citation impact, while the purple rims highlight countries with high betweenness centrality. As shown in Figure 3D, the United States, China, Italy, Germany, France, Poland, South Korea, Australia, and Japan exhibit both large node sizes and pronounced purple rings, indicating that they not only contribute substantial output but also serve as bridging actors connecting disparate regions. Other countries, including India, Brazil, Saudi Arabia, and several smaller European nations, form peripheral yet increasingly connected nodes. Taken together, Figure 3A–D show that research on dietary polyphenols and chronic disease prevention is dominated by a small group of high‐income countries but is progressively incorporating emerging economies through an expanding web of international collaborations.

### 3.3 Institutional Collaboration Network and Leading Research Hubs

**FIGURE 4:**
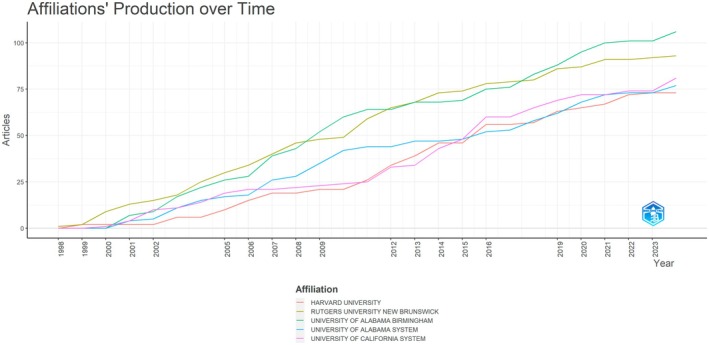
Cumulative publication output of the five most productive affiliations over time.

Institutional‐level analyses revealed a concentrated yet highly interconnected set of research hubs. The temporal trajectories of the five most productive affiliations are presented in Table 3 and Figure 4. As shown in Figure 4, all top institutions are located in the United States, underscoring the central role of North American academic and research organizations in this field. The University of Alabama at Birmingham and the University of Alabama System show an early and steady accumulation of publications from the late 1990s onwards, reflecting a long‐standing interest in dietary polyphenols and chronic disease. Harvard University and Rutgers University–New Brunswick display a slightly later but sharper rise, with particularly pronounced growth after 2005. The University of California System exhibits the most sustained increase, reaching the highest cumulative output by the end of the study period. Together, these trajectories suggest that leading US universities have acted as persistent engines of publication over more than two decades rather than contributing through short‐lived surges.

**TABLE 3:** Leading institutional clusters and their characteristic roles in dietary polyphenol–NCD research.

Institutional cluster	Typical organizations	Characteristic role in the network
US comprehensive universities and systems	University of California System; University of Alabama System; Rutgers University System; Harvard University; University of Minnesota System	Long‐term high output; extensive domestic and international co‐authorship; anchor many multi‐center clinical and mechanistic studies
US federal research agencies and cancer centers	National Institutes of Health (NIH); National Cancer Institute (NCI); United States Department of Agriculture (USDA); major cancer institutes	Provide methodological and mechanistic reference work; frequently cited across clusters; act as intellectual and funding hubs
Western European public research organizations	INSERM; CNRS; INRAE; CNR; CSIC and associated biomedical networks	Coordinate multi‐country consortia; bridge nutrition, basic science, and clinical medicine; central in European collaboration subnetwork
European and Latin American universities	University of Porto; University of Barcelona; Universitat Rovira i Virgili; University of Milan; other regional universities	Contribute substantial empirical data; participate in cross‐border collaborations within Europe and with the Americas
Asian universities and medical centers	Zhejiang University; Chang Gung University; China Agricultural University; selected medical universities	Represent expanding participation from Asia; increasingly involved in mechanistic and translational studies, often in partnership with Western institutions

**FIGURE 5:**
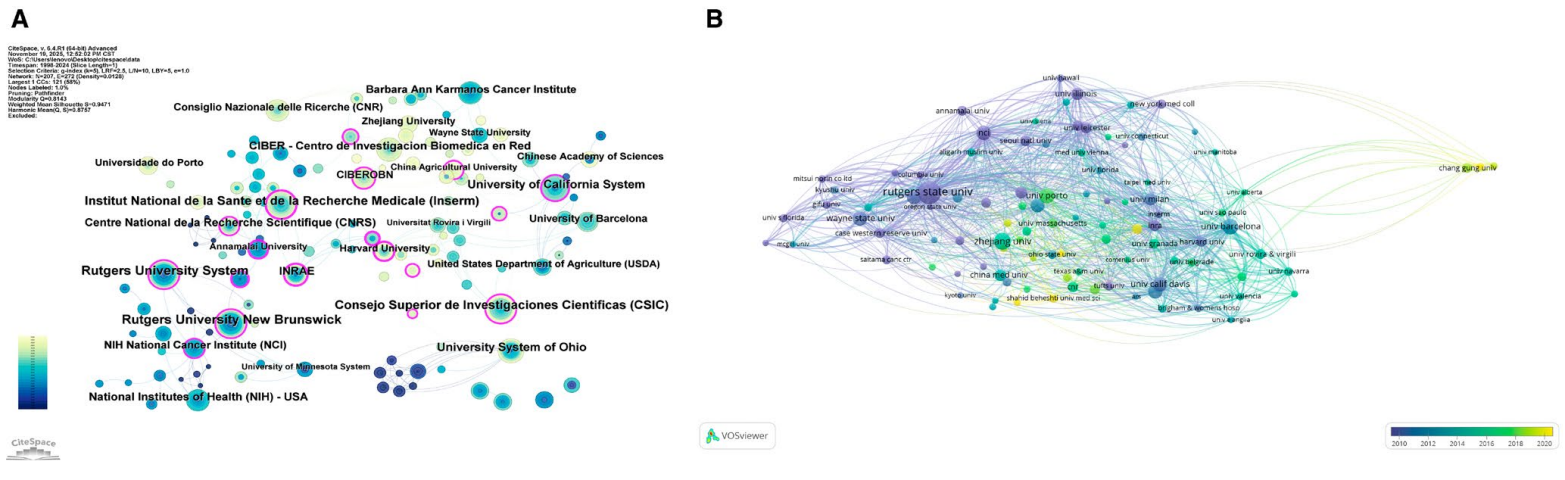
Institutional collaboration and knowledge networks. (A) Institutional co‐authorship network of organizations publishing on dietary polyphenols and NCDs. (B) Institutional co‐citation network depicting the main research hubs and their linkages.

Beyond individual institutions, the VOSviewer collaboration network illustrates how research organizations are embedded in a dense web of partnerships (Figure 5A). As shown in Figure 5A, the network is dominated by several large nodes, including Rutgers State University, Zhejiang University, the University of Porto, the University of Barcelona, the University of Milan, INSERM, INRAE, and multiple campuses of the University of California. These institutions maintain extensive co‐authorship ties with one another and with numerous smaller universities and research centers across Europe, North America, Asia, and Latin America. The color gradient, which reflects the average publication year, indicates that certain institutions, such as Zhejiang University and Chang Gung University, have become increasingly prominent in more recent years, expanding the network beyond its original North American and Western European core. Distinct but overlapping clusters can be discerned that correspond broadly to European consortia (e.g., the University of Porto, University of Barcelona, University of Milan, INRAE, and INSERM), North American universities and cancer centers, and an emergent Asian cluster anchored by Chinese and Taiwanese universities.

The co‐citation network provides complementary insight into which institutions form the intellectual backbone of the field. As shown in Figure 5B, large and highly cited nodes include the National Institutes of Health (NIH) and its National Cancer Institute (NCI), the United States Department of Agriculture (USDA), Harvard University, the University of California System, Rutgers University System, Consejo Superior de Investigaciones Científicas (CSIC), Consiglio Nazionale delle Ricerche (CNR), the French national research organizations (INSERM and CNRS), and several major European universities. The presence of thick purple rings around nodes such as NIH, NCI, Rutgers University System, CSIC, INRAE, and the University of California System indicates high betweenness centrality, meaning that these institutions stand at key junctions in the flow of knowledge and frequently connect otherwise separate groups of citing and cited organizations.

When the collaboration and co‐citation structures are considered together (Figure 5A,B), a coherent picture emerges of a multi‐centered but tightly linked research ecosystem. A relatively small set of universities and public research agencies not only produce a large share of the primary literature but also constitute the main intellectual reference points for subsequent work. At the same time, numerous medium‐sized institutions—such as the University of Porto, Annamalai University, the University of Barcelona, and various state universities in North America and Europe—participate in these networks, suggesting that the field is sustained by broad‐based collaboration rather than by a handful of isolated centers.

### 3.4 Journal Landscape and Underlying Knowledge Base

**FIGURE 6:**
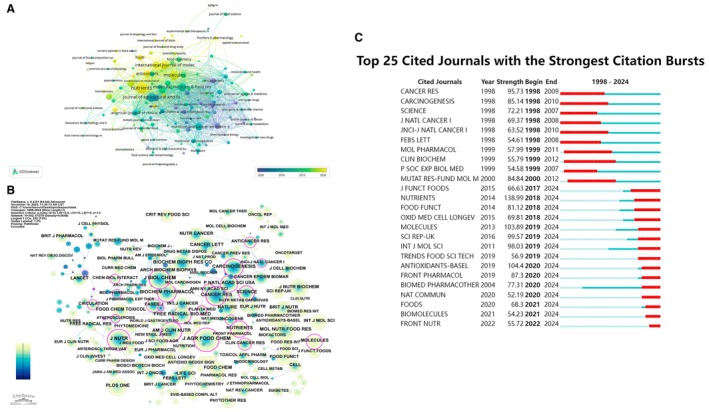
Core journal networks and evolution of the knowledge base. (A) Source journal co‐occurrence network in dietary polyphenol–NCD research. (B) Cited‐journal co‐citation network representing the underlying knowledge base. (C) Top 25 cited journals with the strongest citation bursts, 1998–2024.

The 3280 articles were distributed across a broad spectrum of journals, but publication activity was concentrated in a limited number of titles. The source journal network constructed with VOSviewer is shown in Figure 6A. As shown in Figure 6A, several nutrition and food science journals occupy central positions with large node sizes and dense linkages, including Nutrients, Molecules, Journal of Agricultural and Food Chemistry, Molecular Nutrition & Food Research, Antioxidants, and Nutrition and Cancer. These journals form the core of a tightly interconnected cluster that links food chemistry, nutritional epidemiology, and mechanistic studies. Surrounding this core are journals specializing in oncology (e.g., Cancer Letters, Cancer Research), pharmacology and toxicology, and general biochemistry and molecular biology. The color gradient, reflecting average publication year, suggests a shift over time from traditional cancer and biochemistry outlets toward newer open‐access nutrition and food science journals, particularly after 2010.

Co‐citation analysis further clarified the journals that constitute the intellectual foundation of the field. The CiteSpace map of co‐cited journals (Figure 6B) reveals a dense structure in which a relatively small subset of titles accounts for a large proportion of co‐citation links. As shown in Figure 6B, *Journal of Nutrition, American Journal of Clinical Nutrition*, *Journal of Agricultural and Food Chemistry*, *Free Radical Biology and Medicine*, *Biochemical Pharmacology*, *Cancer Research*, *Carcinogenesis*, and leading general‐science journals such as *Science*, *Nature*, and *The Lancet* form the backbone of the network. Journals with prominent purple rims—indicating high betweenness centrality—include *Journal of Nutrition*, *Journal of Agricultural and Food Chemistry*, *Free Radical Biology and Medicine*, *Nutrition Research*, and *Food Chemistry*, highlighting their bridging role between nutrition, food science, basic biochemistry, and clinical medicine. These patterns indicate that research on dietary polyphenols and non‐communicable diseases draws simultaneously on the traditions of nutritional sciences, redox biology, and cancer biology.

Temporal changes in the journal‐level knowledge base are summarized by the burst‐detection analysis of cited journals (Figure 6C). As shown in Figure 6C, early bursts (late 1990s to around 2010) were dominated by oncology and basic science journals, including *Cancer Research*, *Carcinogenesis*, *Journal of the National Cancer Institute*, *FEBS Letters*, *Molecular Pharmacology*, and *Clinical Biochemistry*, indicating that initial work on polyphenols and chronic disease prevention was anchored in experimental oncology and molecular biology. From approximately 2015 onwards, strong and sustained bursts emerged for nutrition‐ and food‐focused journals such as *Journal of Functional Foods*, *Nutrients*, *Food & Function*, *Oxidative Medicine and Cellular Longevity*, *Molecules*, *Scientific Reports*, *Trends in Food Science & Technology*, *Antioxidants*, *Frontiers in Pharmacology*, *Foods*, *Biomolecules*, and *Frontiers in Nutrition*. These recent bursts underline the progressive institutionalization of polyphenol research within dedicated nutrition and food science outlets and the growing visibility of open‐access multidisciplinary platforms.

**FIGURE 7:**
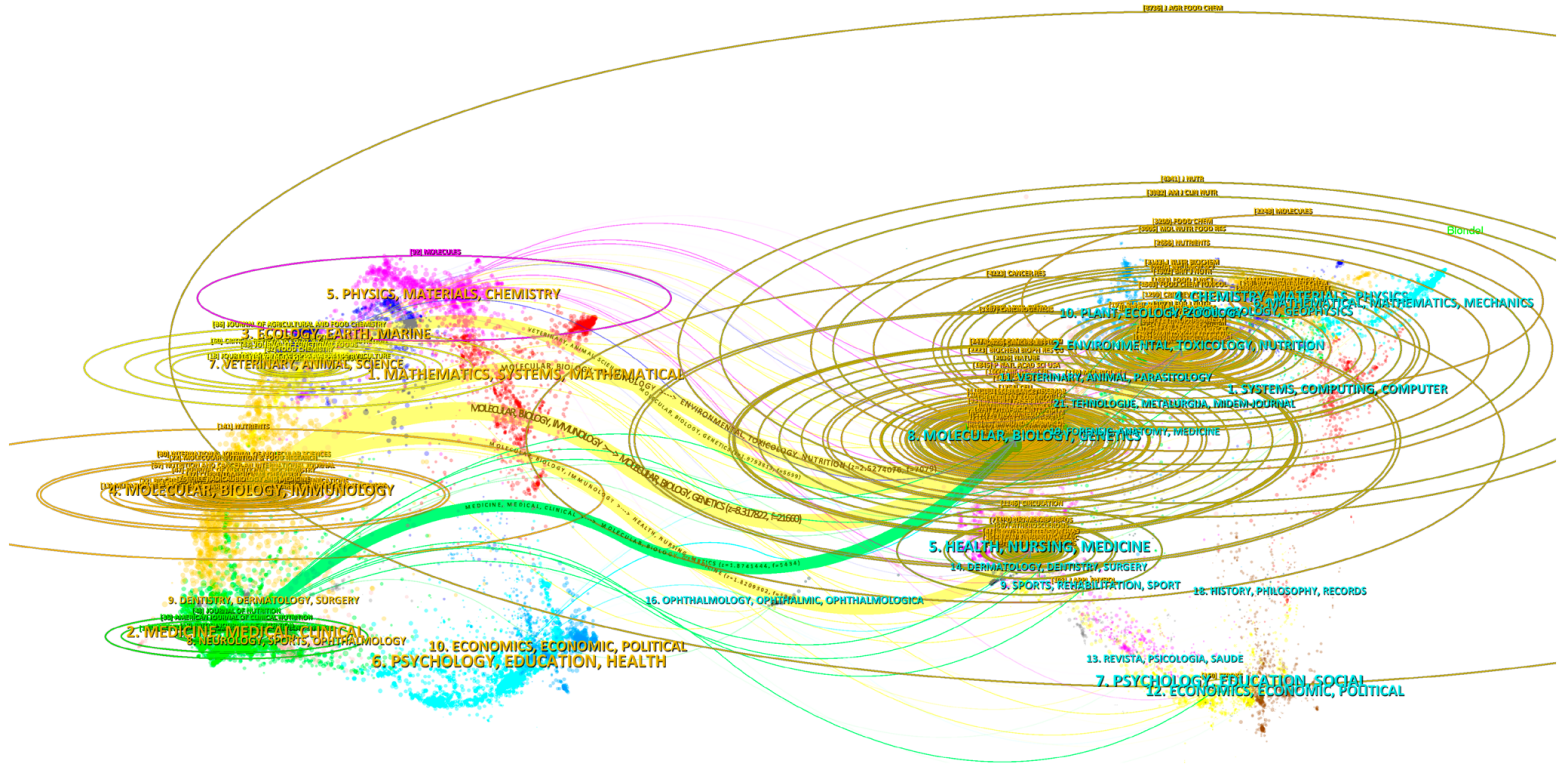
Dual‐map overlay of citing and cited journals in dietary polyphenol–NCD research.

The dual‐map overlay of journals (Figure 7) provides a synoptic view of how citing and cited disciplines are connected. As shown in Figure 7, most citing articles are located in clusters labeled “medicine, medical, clinical”, “molecular biology, immunology”, and “nutrition, sports, ophthalmology”, as well as “environmental, toxicology, nutrition”. The main citation trajectories originate from these clusters and converge on cited journals in the domains of “molecular biology, genetics”, “health, nursing, medicine”, and “food science, toxicology, nutrition”, indicating that polyphenol–NCD research operates at the interface of clinical medicine, molecular mechanisms, and food science. Table 4 groups representative journals into disciplinary families based on these patterns, highlighting the layered structure of the publication venues.

**TABLE 4:** Representative journals and disciplinary families in dietary polyphenol–NCD research.

Disciplinary family	Representative source and cited journals	Characteristic contribution to the field
Nutrition and dietetics	*Nutrients*; *Journal of Nutrition*; *American Journal of Clinical Nutrition*; *European Journal of Nutrition*; *Frontiers in Nutrition*	Publish epidemiological and clinical studies relating dietary polyphenol intake to NCD risk and progression
Food science and agricultural chemistry	*Journal of Agricultural and Food Chemistry*; *Food Chemistry*; *Journal of Functional Foods*; *Food & Function*; *Trends in Food Science & Technology*; *Foods*	Focus on polyphenol characterization, food matrices, bioavailability, and functional food development
Basic biochemistry, redox biology, and molecular sciences	*Journal of Biological Chemistry*; *Free Radical Biology and Medicine*; *Biochemical Pharmacology*; *FEBS Letters*; *Biochemical and Biophysical Research Communications*; *Oxidative Medicine and Cellular Longevity*; *International Journal of Molecular Sciences*; *Molecules*; *Antioxidants*	Provide mechanistic insights into oxidative stress, inflammation, signaling pathways, and molecular targets of polyphenols
Oncology and cancer prevention	*Cancer Research*; *Carcinogenesis*; *Cancer Letters*; *International Journal of Cancer*; *Nutrition and Cancer*; *Oncotarget*	Address chemopreventive and adjuvant roles of polyphenols in cancer initiation, promotion, and progression
Multidisciplinary and general science	*Science*; *Nature*; *Nature Communications*; *PLOS ONE*; *Scientific Reports*	Offer broad dissemination for high‐impact or cross‐disciplinary studies, often linking polyphenols to genomics, systems biology, or public health

### 3.5 Core Topics and High‐Frequency Terms in Polyphenol–NCD Research

High‐frequency keywords provide a concise summary of the dominant scientific concerns in this field. As shown in Figure 2A, the 10 most frequent Keywords Plus terms are “in vitro”, “oxidative stress”, “green tea”, “inhibition”, “expression”, “NF‐kappa‐B”, “apoptosis”, “red wine”, “cancer”, and “polyphenols”. Experimental context (“in vitro”) and mechanistic concepts (“oxidative stress”, “NF‐kappa‐B”, “apoptosis”, “expression”, “inhibition”) clearly dominate, indicating that much of the literature has focused on cellular and molecular pathways rather than solely on clinical endpoints. The prominence of specific exposures such as “green tea”, “red wine”, and “polyphenols” reflects the central role of tea catechins, wine‐derived resveratrol, and mixed polyphenol mixtures as model systems for exploring chronic disease prevention. The word cloud derived from all high‐frequency terms (Figure 2B) reinforces this impression. In addition to the leading terms listed above, Figure 2B highlights “antioxidant activity”, “insulin‐resistance”, “coronary‐heart‐disease”, “(−)‐epigallocatechin gallate”, “quercetin”, and “trans‐resveratrol”, underscoring the dual focus on redox and inflammatory mechanisms and on major non‐communicable chronic diseases such as cardiovascular disease, diabetes, and cancer.

**FIGURE 8:**
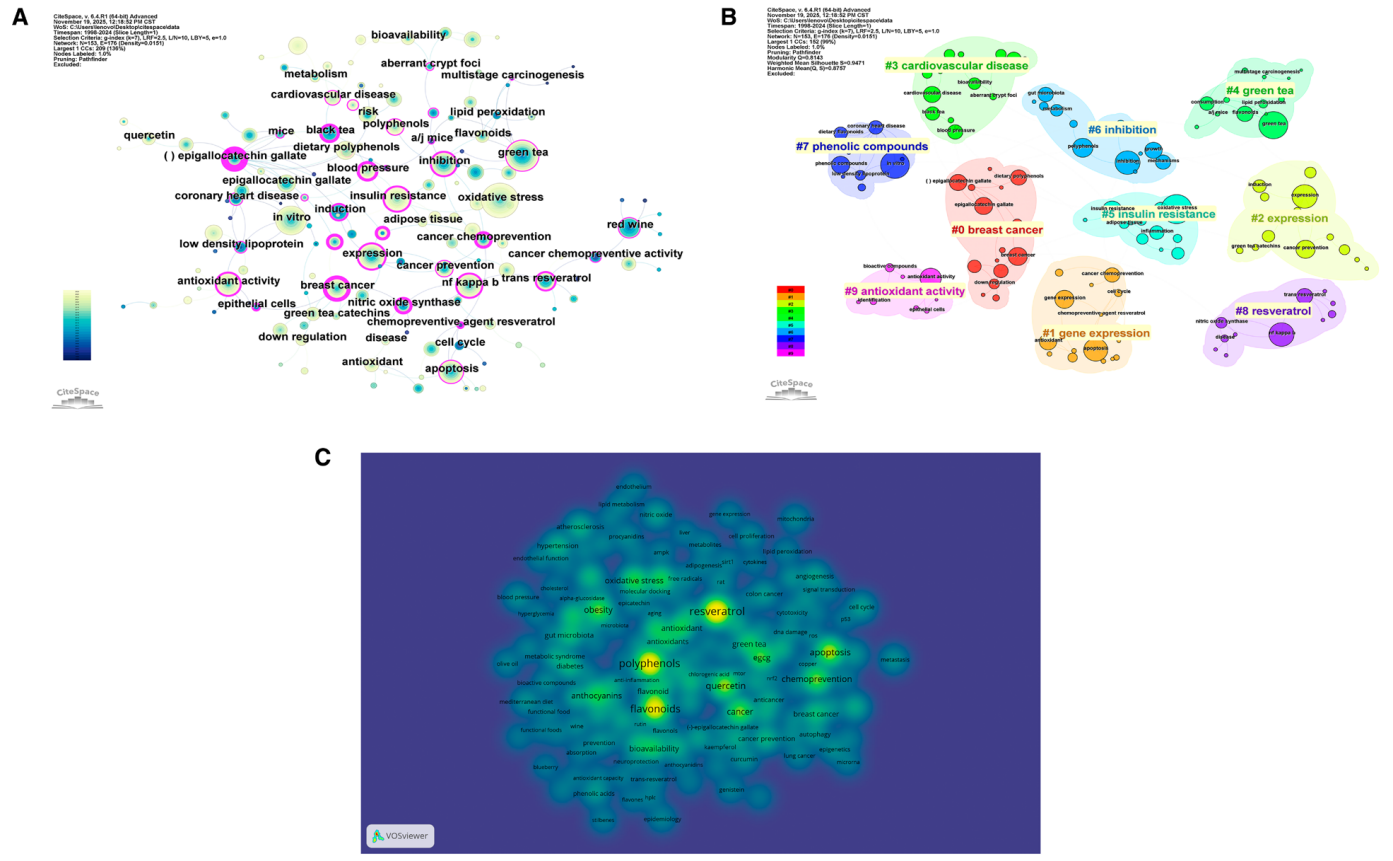
Co‐occurrence Network Structure and Hotspot Distribution of Research Themes. (A) Keyword co‐occurrence network showing major concepts in the field. (B) Keyword clustering map identifying principal thematic groups. (C) Keyword density (spectrum) map highlighting hotspots of research attention.

The structure of relationships among these terms is depicted in the CiteSpace keyword co‐occurrence network (Figure 8A). As shown in Figure 8A, central nodes include “dietary polyphenols”, “in vitro”, “oxidative stress”, “insulin resistance”, “cardiovascular disease”, “cancer prevention”, “breast cancer”, “epigallocatechin gallate”, “resveratrol”, and “red wine”. Thick links connect mechanistic terms to specific outcomes: for example, “oxidative stress” and “lipid peroxidation” are closely associated with “cardiovascular disease”, “low density lipoprotein”, and “blood pressure”; “apoptosis”, “cell cycle”, and “nitric oxide synthase” connect to “breast cancer” and “cancer chemoprevention”; and “insulin resistance” and “adipose tissue” link to “inflammation” and “metabolism”. Keywords with purple rims, such as “oxidative stress”, “insulin resistance”, “antioxidant activity”, and “epigallocatechin gallate”, exhibit high betweenness centrality, suggesting that they bridge multiple thematic areas and function as integrative concepts across the literature.

Cluster analysis of the co‐occurrence network identifies 10 major thematic domains (Figure 8B). As shown in Figure 8B, the largest cluster (#0 “breast cancer”) groups terms related to mammary carcinogenesis, cell‐cycle control, and chemopreventive agents such as epigallocatechin gallate and resveratrol. Cluster #1 (“gene expression”) encompasses apoptosis, signaling pathways, and transcriptional regulation, capturing mechanistic studies that explore how polyphenols modulate cellular programs. Cluster #2 (“expression”) is closely related but emphasizes experimental read‐outs for signaling molecules and transcription factors. Cluster #3 (“cardiovascular disease”) brings together “blood pressure”, “low‐density lipoprotein”, “bioavailability”, and “back tea/black tea”, reflecting work on vascular function and atherogenesis. Cluster #4 (“green tea”) focuses on tea catechins and their chemopreventive effects, whereas cluster #5 (“insulin resistance”) covers obesity, adipose tissue, inflammation, and glucose homeostasis. Cluster #6 (“inhibition”) aggregates general terms relating to suppression of growth, proliferation, and carcinogenesis; cluster #7 (“phenolic compounds”) centers on in vitro characterization of polyphenolic mixtures; cluster #8 (“resveratrol”) on stilbene‐specific mechanisms; and cluster #9 (“antioxidant activity”) on the measurement and biological consequences of antioxidant capacity. These clusters and their representative terms are summarized qualitatively in Table 5.

**TABLE 5:** Major keyword clusters and their thematic interpretation.

Cluster label	Representative keywords	Theme
#0 Breast cancer	Breast cancer; epigallocatechin gallate; resveratrol; chemopreventive agent; down regulation	Polyphenol‐mediated chemoprevention and treatment of breast cancer
#1 Gene expression	Gene expression; apoptosis; cell cycle; antioxidant; chemoprevention	Modulation of transcriptional programs, apoptosis, and cell‐cycle control by polyphenols
#2 Expression	Expression; induction; green tea catechins; cancer prevention	Experimental read‐outs of signaling pathways in response to polyphenol exposure
#3 Cardiovascular disease	Cardiovascular disease; blood pressure; low‐density lipoprotein; bioavailability; black tea	Vascular and lipid‐related mechanisms underlying cardioprotective effects
#4 Green tea	Green tea; flavonoids; lipid peroxidation; multistage carcinogenesis	Chemopreventive and antioxidant actions of tea polyphenols
#5 Insulin resistance	Insulin resistance; adipose tissue; inflammation; oxidative stress	Effects of polyphenols on obesity, insulin sensitivity, and metabolic inflammation
#6 Inhibition	Inhibition; growth; mechanisms; polyphenols	General inhibitory effects on tumor growth and pathological processes
#7 Phenolic compounds	Phenolic compounds; in vitro; dietary flavonoids; low density lipoprotein	In vitro characterization and functional evaluation of phenolic mixtures
#8 Resveratrol	Resveratrol; trans‐resveratrol; NF‐kappa‐B; nitric oxide synthase	Stilbene‐specific pathways involving inflammatory and endothelial targets
#9 Antioxidant activity	Antioxidant activity; bioactive compounds; identification; epithelial cells	Assessment of antioxidant capacity and related biological actions

The temporal evolution of these themes is illustrated in the keyword “mountain” plot (Figure S1). As shown in Figure S1, clusters such as “breast cancer”, “gene expression”, “expression”, and “cardiovascular disease” display a broad and persistent presence from the late 1990s to 2024, indicating stable long‐term interest. In contrast, “insulin resistance”, “phenolic compounds”, “resveratrol”, and “antioxidant activity” show relatively more pronounced peaks in the mid‐to‐late 2000s and 2010s, suggesting that metabolic disease, compound‐focused studies, and refined antioxidant assays gained prominence somewhat later. “Green tea” maintains a sustained though slightly declining profile, consistent with the early dominance of tea‐based research followed by diversification into other dietary sources and compounds. Together, the frequency distributions, co‐occurrence patterns, and temporal profiles demonstrate that research on dietary polyphenols and NCDs is organized around a set of interconnected mechanistic and disease‐oriented themes, with enduring attention to redox biology and cancer prevention and an expanding focus on cardiometabolic disorders and specific bioactive compounds.

### 3.6 Keyword Co‐Occurrence Structure and Thematic Clusters

The spatial organization of high‐frequency terms is illustrated in the VOSviewer density map (Figure 8C). As shown in Figure 8C, four dense cores dominate the conceptual landscape. The first core is centered on generic exposure terms—“polyphenols”, “flavonoids”, “anthocyanins”—and methodological descriptors such as “bioavailability”, reflecting a broad interest in chemical classes and pharmacokinetic behavior. A second core aggregates specific compounds and sources, including “resveratrol”, “quercetin”, “EGCG”, and “green tea”, indicating that a limited set of model foods and molecules anchor much of the experimental and clinical work. A third core comprises disease‐related keywords such as “obesity”, “diabetes”, “metabolic syndrome”, “atherosclerosis”, “hypertension”, and “breast cancer”, suggesting that cardiometabolic disorders and hormone‐dependent cancers remain the principal clinical targets. The fourth core is dominated by mechanistic and pathway‐related terms—“oxidative stress”, “apoptosis”, “chemoprevention”, “gut microbiota”, “angiogenesis”, “epigenetics”—which surround and connect the exposure and disease clusters. The gradual change in density between these cores underlines the continuity between compound‐oriented, mechanistic, and disease‐oriented studies rather than strict compartmentalisation. The detailed structure of co‐occurrence relations is captured by the CiteSpace keyword network (Figure 8A). As shown in Figure 8A, the network exhibits a mixed hub‐and‐cluster architecture: a few central terms, such as “dietary polyphenols”, “in vitro”, “insulin resistance”, and “cardiovascular disease”, are connected to many peripheral nodes, whereas more specialized terms (e.g., “aberrant crypt foci”, “multistage carcinogenesis”, “adipose tissue”) form smaller satellite constellations. Edges linking disease terms to mechanistic descriptors (e.g., connections between “coronary heart disease” and “lipid peroxidation” or between “breast cancer” and “apoptosis”) suggest that studies typically articulate both a clinical endpoint and one or more biological pathways. The presence of multiple short paths between distinct disease areas indicates that mechanistic concepts such as oxidative stress or inflammation serve as shared explanatory frameworks across cardiovascular, metabolic, and oncologic research, providing structural coherence to the network.

Thematic clustering of keywords delineates this structure into 10 coherent communities (Figure 8B). As shown in Figure 8B, clusters related to breast cancer (#0), gene expression (#1), and expression‐related read‐outs (#2) lie in close proximity, forming a contiguous “oncology–molecular biology” region. In contrast, the cardiovascular disease cluster (#3) is positioned between tea‐focused cluster #4 and the more general inhibition cluster (#6), reflecting a gradient from specific exposures to broader mechanistic work on vascular and lipid endpoints. The insulin resistance cluster (#5) occupies an intermediate location between oxidative‐stress terminology and obesity‐related descriptors, highlighting the central role of metabolic inflammation as a bridge between adiposity and chronic disease outcomes. Clusters devoted to phenolic compounds (#7), resveratrol (#8), and antioxidant activity (#9) are situated toward the periphery but maintain multiple links to the central disease clusters, indicating that compound‐centric and assay‐focused studies are conceptually anchored in clinical questions rather than entirely methodological. The geometry of the map thus reveals a layered thematic structure in which exposure, mechanism, and disease clusters overlap and interpenetrate rather than existing as separate strands.

The institutional collaboration network provides an external validation of this thematic arrangement. As shown in Figure 5A, institutions are organized into overlapping sub‐networks rather than isolated blocks, with dense regions corresponding to long‐standing consortia and looser regions representing newer or more specialized centers. When juxtaposed with the keyword clusters (Figure 8A,B), the multi‐hub nature of Figure 5A suggests that major institutions simultaneously contribute to several thematic communities: groups that are central in the collaboration network are also those likely to work across cancer, cardiometabolic disease, and mechanistic investigations. Peripheral institutions, by contrast, appear to occupy niche positions that map onto specific thematic islands, such as resveratrol‐focused oncology or antioxidant‐assay development. This convergence of semantic and collaborative structures indicates that thematic diversity in polyphenol–NCD research is sustained not by isolated specialist groups, but by interlocking institutional networks that bridge multiple disease areas and mechanistic paradigms.

### 3.7 Temporal Evolution of Research Themes and Emerging Hotspots

**FIGURE 9:**
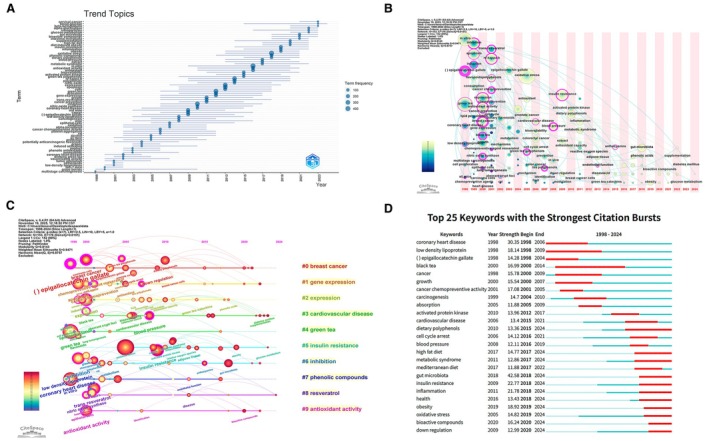
Temporal Evolution and Frontier Emergence of Research Themes. (A) Trend topics over time based on high‐frequency terms, 1998–2024. (B) Keyword timezone map showing the sequential emergence of major topics. (C) Timeline view of keyword clusters in dietary polyphenol–NCD research, 1998–2024. (D) Top 25 keywords with the strongest citation bursts in dietary polyphenol–NCD research, 1998–2024.

To characterize how scientific attention has shifted over the past quarter‐century, we examined the temporal distribution of high‐frequency terms and clusters. As shown in Figure 9A, the trend‐topic analysis reveals a clear sequence of thematic waves. In the earliest years (1998–2003), the most prominent terms were “multistage carcinogenesis”, “tumor promotion”, “coronary heart disease”, “low‐density lipoproteins”, “drinking green tea”, and “grape berries”. These early topics reflect a strong focus on classical chemoprevention models and lipid‐related cardiovascular endpoints, often centered on specific beverages such as green tea and red wine. Between 2004 and 2012, the vocabulary broadened toward signaling pathways and cellular responses—terms such as “nitric‐oxide synthase”, “NF‐kappa‐B”, “apoptosis”, “oxidative stress”, and “epithelial cells” appear with increasing frequency—indicating consolidation of mechanistic research that linked dietary polyphenols to redox and inflammatory cascades. From approximately 2013 onwards, newer concepts gain prominence, including “metabolic syndrome”, “high fat diet”, “gut microbiota”, “alpha‐glucosidase”, and “intestinal microflora”, accompanied by disease‐specific terms such as “diabetes mellitus” and “obesity”. This pattern points to a gradual shift from compound‐centered and cancer‐focused work toward integrative studies of metabolic health and host–microbe interactions.

The keyword timezone map provides a complementary, more fine‐grained view of this evolution (Figure 9B). As shown in Figure 9B, terms associated with tea catechins and classical antioxidant paradigms (“green tea”, “epigallocatechin gallate,” “black tea,” “antioxidant activity”) dominate the left‐hand side of the map, indicating their early entrance and long persistence. Around the mid‐2000s, cardiovascular and vascular‐related expressions (“cardiovascular disease”, “blood pressure”, “endothelial function”) emerge and progressively move toward the center of the timeline, suggesting that vascular outcomes became a major focus once fundamental antioxidant mechanisms had been established. More recent years on the right‐hand side are characterized by the appearance of “metabolic syndrome”, “anthocyanins”, “phenolic acids”, “gut microbiota”, “bioactive compounds”, “supplementation”, and “diabetes mellitus”. The sequential arrangement of these terms underscores a transition from single‐compound models to broader dietary patterns and complex phenotypes involving energy metabolism, microbiome composition, and long‐term cardiometabolic health.

The timeline view of keyword clusters further clarifies how individual thematic communities evolve (Figure 9C). As shown in Figure 9C, the “breast cancer” cluster (#0) dominates the earliest phase with large nodes for “epigallocatechin gallate”, “down regulation”, and “chemopreventive agents”, but its relative prominence diminishes after approximately 2010, even though the cluster remains active. In contrast, the “insulin resistance” cluster (#5) begins modestly but shows a marked intensification from 2010 onwards, with large nodes for “metabolic syndrome”, “high fat diet”, “obesity”, “glucose metabolism”, and “gut microbiota” appearing in the last decade. The “cardiovascular disease” cluster (#3) spans the entire timeline, but its later nodes increasingly feature terms such as “phenolic acids”, “supplementation”, and “Mediterranean diet”, suggesting a move from mechanistic vascular studies toward dietary‐pattern interventions. Clusters devoted to “phenolic compounds” (#7), “resveratrol” (#8), and “antioxidant activity” (#9) show peak activity in the mid‐2000s, after which they continue at a lower intensity, indicating that compound‐specific discovery has largely given way to integrative translational research.

Emerging hotspots are captured by the burst‐detection analysis of keywords (Figure 9D). As shown in Figure 9D, early high‐intensity bursts correspond to “coronary heart disease”, “low density lipoprotein”, “(−)‐epigallocatechin gallate”, “black tea”, “cancer”, and “carcinogenesis”, confirming that lipid metabolism and carcinogenesis were the initial focal points. During the intermediate period, bursts shift toward mechanistic markers and vascular outcomes, including “absorption”, “oxidative stress”, “activated protein kinase”, “cell cycle arrest”, “cardiovascular disease”, and “blood pressure”, reflecting intense interest in signaling pathways and endothelial biology. From roughly 2017 onwards, new bursts appear for “high fat diet”, “metabolic syndrome”, “Mediterranean diet”, “gut microbiota”, “inflammation”, “health”, “obesity”, “bioactive compounds”, and “down regulation”. Many of these bursts extend up to 2024, indicating that metabolic health, lifestyle‐related obesity, and microbiome‐mediated mechanisms currently constitute the most dynamic research fronts. Taken together, Figure 9A–D delineate a coherent temporal trajectory whereby polyphenol research has progressed from early antioxidant and cancer chemoprevention models to a mature, multidimensional agenda encompassing cardiometabolic diseases, dietary patterns, and host–microbe interactions.

## 4 Discussion

Through the use of comprehensive bibliometric and science‐mapping techniques, this metadocumentary study shows that research on dietary polyphenols in relation to NCDs has evolved into a mature, globally networked field with distinct thematic and geographical structures. Rather than a collection of isolated compound‐ or disease‐specific investigations, the literature now forms an interconnected ecosystem linking polyphenol‐rich foods and extracts, oxidative and inflammatory pathways, cardiometabolic and oncologic endpoints, and an increasingly prominent microbiota–metabolism axis (Halagali et al. 2025; Macena et al. 2022; Kiyimba et al. 2023). At the same time, the dominance of mechanistic in vitro and animal work, the uneven global distribution of research capacity, and the concentration of attention on a subset of NCDs and polyphenol sources underscore that this field is still some distance from delivering the kind of human‐relevant, context‐sensitive evidence base needed for policy and clinical decision‐making (Halagali et al. 2025).

The temporal pattern of publications suggests that dietary polyphenol–NCD research has largely followed the trajectory described by Price's model of scientific growth, with an initial formative period, a phase of rapid expansion, and a subsequent transition to a high‐volume but more stable stage of development. Early years were characterized by small but steadily increasing numbers of papers focused on antioxidant properties and classical chemoprevention models. The subsequent rise in annual output and cumulative publications, together with diversification of topics, reflects consolidation of polyphenols as a mainstream subject within nutritional and biomedical research rather than a niche interest. The leveling‐off of annual publication growth while maintaining a high absolute volume is indicative of a mature yet still active domain, in which incremental mechanistic refinements and extensions to new disease models coexist with attempts at translation into human studies (Zelicha et al. 2022; Davinelli et al. 2025; González et al. 2024). The chronological analyses of high‐frequency terms further illustrate that, rather than being replaced, earlier lines of work on oxidative stress and cancer chemoprevention persist as a foundational stratum onto which newer themes—such as metabolic syndrome, gut microbiota and polyphenol‐rich dietary patterns—have been layered (Zelicha et al. 2022).

The global collaboration maps reveal that this expansion has not been geographically uniform. Countries in North America, Western Europe, and East Asia dominate both the volume of publications and the central positions in co‐authorship and co‐citation networks. These regions combine longstanding investments in food, nutrition, and biomedical research infrastructure with high NCD burdens, well‐developed analytical facilities for phytochemicals, and established funding streams for basic and translational research. The increasing contribution of Brazil, India, Iran, and other emerging economies suggests that research capacity in polyphenols and NCDs is broadening, and that locally important crops and dietary patterns are gradually entering the international literature. However, representation from sub‐Saharan Africa and some parts of South‐East Asia remains limited, despite the rapid rise of NCDs in these regions. This under‐representation likely reflects constraints in research funding, laboratory infrastructure, long‐term cohort platforms, and opportunities for international collaboration, rather than a lower public health relevance of polyphenol‐rich foods. Addressing this imbalance will require targeted capacity‐building programs, equitable partnerships with established research hubs, and open approaches to data, methods, and materials that reduce barriers to entry for resource‐constrained settings.

The co‐occurrence and clustering of keywords delineate a field organized around several interlocking thematic domains rather than isolated subtopics. One axis connects specific polyphenol classes and food sources—such as catechins in green tea, resveratrol in red wine and grapes, and mixed phenolics in fruits and cocoa—to mechanistic frameworks centered on oxidative stress, inflammatory signaling, apoptosis and endothelial function (Jia et al. 2024). A second axis links these mechanisms to disease‐focused communities, focusing predominantly on breast and other hormone‐dependent cancers, cardiovascular disease, hypertension, dyslipidaemia, obesity and insulin resistance (Paunovic et al. 2024). A third, more recent axis involves gut microbiota, high‐fat diet models, metabolic syndrome and related cardiometabolic outcomes, reflecting growing interest in host–microbe interactions and energy balance (Pan et al. 2025). The presence of shared mechanistic nodes (e.g., “oxidative stress”, “inflammation”, “NF‐κB”, “insulin resistance”) with high centrality indicates that these concepts serve as common currency across diverse disease models (Zheng et al. 2022; Han et al. 2023; Alves‐Santos et al. 2020), providing an overarching explanatory framework that strengthens the internal coherence of the field but may also risk over‐simplifying complex causal pathways.

The temporal analyses of keyword clusters highlight a clear sequence in the evolution of research priorities. Early emphasis on cancer chemoprevention and lipid‐related cardiovascular outcomes, often in the context of green tea and red wine, is consistent with historical interest in antioxidant vitamins and the “French paradox”. Subsequent years saw a shift toward detailed interrogation of intracellular signaling cascades, endothelial function and vascular biology, as evidenced by the emergence of terms related to nitric oxide synthase, MAP kinases, cell‐cycle control and apoptosis. In the last decade, attention has increasingly turned to metabolic health, with clusters built around obesity, insulin resistance, high‐fat diet models, metabolic syndrome and gut microbiota. It is worth further pointing out that the paradigm shift in research topics from “antioxidant” to “gut microbiota” is not just a natural replacement of theoretical hotspots, but is largely driven and achieved by revolutionary advances in research methodology. Early research relied on measuring biochemical indicators such as total antioxidant capacity, which had a relatively low technical threshold but was difficult to reveal complex biological effects in vivo. In the past decade, the popularization and cost reduction of high‐throughput sequencing technology, especially the maturity of metagenomics and non targeted metabolomics methods, have enabled researchers to systematically and finely depict the composition and function of gut microbiota, and track the complex product spectrum of dietary polyphenols through microbial metabolism and transformation. These technologies provide unprecedented tools to transform ‘gut microbiota’ from a vague concept into a key research dimension that can be quantitatively measured and mechanistically analyzed. This progression suggests a gradual broadening of scope from single‐organ or single‐endpoint models to integrated metabolic phenotypes, and from individual compounds to whole‐diet and host‐microbe perspectives (Jin‐Feng et al. 2025; Quesada‐Vázquez et al. 2024; Dominguez et al. 2021; Kaufman‐Shriqui et al. 2022). The persistence of cancer‐ and vascular‐focused clusters alongside newer metabolic ones underscores that the field has expanded rather than pivoted wholesale, which is appropriate given the multifaceted contribution of polyphenols to different stages and types of NCDs. At the same time, the relative scarcity of keywords related to chronic kidney disease, neurodegenerative disorders or multimorbidity suggests that important areas of NCD burden remain comparatively under‐explored.

The journal‐level analyses reinforce the picture of a field situated at the interface of multiple disciplines. The most productive and most frequently cited journals span nutrition and dietetics, food science and agricultural chemistry, redox biology, molecular pharmacology, oncology and general biomedical sciences. This distribution reflects the inherently translational nature of polyphenol research, which spans from characterization of compounds in plant matrices, through bioavailability and metabolic fate, to clinical and epidemiological investigations of NCD outcomes. The shift in citation bursts from traditional oncology and basic science journals toward newer, often open‐access, nutrition and food science titles in recent years indicates growing institutionalization of polyphenol–NCD research within specialized outlets and increased visibility among nutrition professionals (Beaglehole et al. 2012; Ballini et al. 2024; Hao et al. 2023). While this diversification is positive for dissemination within relevant communities, it also underscores the need for deliberate cross‐talk between basic, food‐focused and clinical journals to avoid fragmentation of evidence and to ensure that mechanistic and analytical advances are translated into studies and guidelines that matter at the population level.

One of the most striking features of the mapped literature is the predominance of mechanistic work conducted in cell lines and animal models, as reflected by the prominence of “in vitro”, “antioxidant activity”, signaling molecules and pathway markers in the high‐frequency term lists. Due to the serious ethical and practical challenges faced by long‐term intervention trials in the human body, including difficulties in maintaining long‐term dietary compliance, the complexity of setting appropriate placebos, and the difficulty in controlling diverse background dietary and lifestyle confounding factors (Satija et al. 2015). In addition, the bioavailability of polyphenols in the human body varies significantly among individuals, and their effects are often mediated through complex interactions between the host microbiota (Manach et al. 2016; Rowland et al. 2017). Moreover, the long development cycle of chronic disease outcomes makes it extremely difficult to design human trials that are both scientific, feasible, and ethically compliant (Frieden 2017). Therefore, this phenomenon can be understood. However, it also reveals a structural imbalance between the depth of molecular understanding and the breadth of human evidence on clinically meaningful NCD endpoints (Fardet and Rock 2018). Many studies employ concentrations, exposure durations or isolated compounds that are difficult to reconcile with typical dietary intakes, and frequently treat polyphenols as stand‐alone pharmacological agents rather than components of complex food matrices and dietary patterns (Fraga et al. 2019; Rodriguez‐Mateos et al. 2014). Moreover, mechanistic work has traditionally emphasized direct antioxidant effects despite accumulating evidence that modulation of cell signaling, gene expression, microbiota composition and metabolite profiles may be at least as important (Kim et al. 2014; Li et al. 2024). The bibliometric patterns suggest that while conceptual thinking in the field has evolved beyond a simplistic “antioxidant” paradigm, the experimental toolkit and model choice have not always fully kept pace.

The relative scarcity of large, long‐term human intervention trials or prospective cohort analyses that explicitly integrate detailed polyphenol exposure assessment with incident NCD outcomes is another critical gap revealed by this metadocumentary mapping. Within the bibliometric dataset, human work is present but is often concentrated on intermediate biomarkers (e.g., blood pressure, lipids, insulin sensitivity) or short‐term surrogate endpoints, and tends to focus on a limited set of foods (such as tea, cocoa, berries, and wine) and populations in high‐income countries. Trial designs are frequently constrained by challenges in standardizing polyphenol doses, controlling background diet, accounting for inter‐individual differences in metabolism and gut microbiota, and choosing appropriate follow‐up durations. Observational studies, while more reflective of real‐world exposures, are hampered by variability in food composition tables, incomplete coverage of polyphenol subclasses, and potential confounding by overall dietary quality and socio‐economic factors (World Health Organization 2024a; Zamora‐Ros et al. 2014; Ficco et al. 2024). The patterns observed in our analysis therefore underscore the need for harmonized exposure assessment tools, validated intake biomarkers, and closer integration of mechanistic and epidemiological perspectives to enable robust causal inference.

Beyond the balance between mechanistic and human evidence, several conceptual and methodological challenges remain unresolved. The conflation of chemically distinct polyphenols into broad categories, combined with heterogeneous analytical methods, complicates comparison across studies and hampers the identification of structure–function relationships. Synergistic and antagonistic interactions between different polyphenols and between polyphenols and other dietary components are rarely addressed systematically, even though they likely shape biological effects in vivo. Inter‐individual variability in response, including differences in gut microbiota composition, genetic polymorphisms, and co‐medications, is increasingly recognized but not yet routinely incorporated into study design or analysis (Yiwen et al. 2024; Mendis et al. 2025). Furthermore, the literature mapped here gives relatively little attention to life‐course perspectives or to the role of polyphenol‐rich diets in populations experiencing dual burdens of malnutrition and NCDs. Together, these gaps suggest that future research agendas will need to move beyond one‐compound‐one‐outcome models toward designs that explicitly accommodate complexity, heterogeneity, and context.

This study has several strengths. By drawing simultaneously on Web of Science, PubMed/MEDLINE and DOAJ, and by applying rigorous de‐duplication, harmonization and manual curation procedures, we were able to assemble a large, cross‐disciplinary corpus spanning more than two decades and to minimize biases introduced by any single database. The combination of performance indicators, co‐authorship and co‐citation networks, keyword clustering, temporal trend analyses and dual‐map overlays provides a multi‐layered view of the field that would not be attainable through narrative review alone. Treating the literature itself as an object of investigation allowed us to identify structural imbalances, geographic inequities and thematic blind spots that may not be apparent from disease‐ or compound‐specific overviews. However, some limitations should also be acknowledged. Our reliance on three databases and English‐language publications means that relevant work indexed elsewhere or reported in other languages may have been missed, particularly regional journals focusing on local crops or dietary patterns. Citation counts for the most recent years are inevitably truncated, and the use of metadata for keyword and affiliation analyses depends on the completeness and consistency of indexing; despite manual standardization, residual misclassification of authors, institutions or terms cannot be completely excluded. Bibliometric methods also cannot assess the methodological quality, risk of bias or effect sizes of individual studies, nor can they determine causality between polyphenol intake and NCD outcomes. Meanwhile, explain that high‐frequency mechanistic nodes may represent widely adopted experimental models (like NF‐κB signaling) that are tractable for study, which does not equate to them being the sole or most critical in vivo mechanism. Note that the persistence of certain concepts (e.g., the early antioxidant hypothesis) in citation networks reflects their foundational role in shaping the field's history and vocabulary, a dynamic that bibliometric methods capture but cannot qualitatively evaluate. In addition, citation counts can be influenced by factors beyond scientific merit, including a publication's visibility, author prominence, journal reputation, and the self‐reinforcing dynamics of the “Matthew effect,” whereby highly cited works attract further citations independent of quality. Future extensions of this work could integrate additional databases and languages, link bibliometric mapping with systematic evidence evaluation (such as using GRADE framework, etc.) for comprehensive evaluation, and incorporate trial registries, cohort consortia and omics repositories to provide a more granular and dynamic picture of how mechanistic insights translate into actionable human evidence.

The present findings suggest that research on dietary polyphenols and non‐communicable chronic diseases is well‐positioned for a new phase in which mechanistic sophistication is matched by human‐centered, context‐aware study designs and globally inclusive collaborations. Building on the structures identified here, future work should prioritize multi‐centere, long‐term interventions and cohort studies that test realistic polyphenol‐rich dietary patterns in diverse populations; adopt standardized and transparent approaches to exposure assessment, outcome definition and reporting; and embed metabolomics, microbiome profiling and other systems‐level tools to capture inter‐individual variability and mechanistic pathways. At the same time, greater engagement with under‐represented regions, locally important food systems and implementation research will be essential if the promise of polyphenol‐rich diets for NCD prevention is to be realized beyond highly controlled experimental settings.

## 5 Conclusion

Research on dietary polyphenols and non‐communicable chronic diseases has become a leading, globally networked domain in nutrition and chronic disease prevention, driven primarily by teams in the United States, China, Japan, Western Europe, and a growing number of emerging economies. Current scientific attention converges on cardiometabolic and oncologic outcomes, with distinct emphases on oxidative and inflammatory mechanisms, metabolic syndrome, and gut microbiota‐mediated effects depending on the polyphenol classes, food sources, and study designs considered. Most published work remains concentrated in mechanistic in vitro and animal experiments, complemented by observational epidemiology, while relatively few large, long‐term human intervention trials directly test polyphenol‐rich dietary patterns on hard NCD endpoints. Going forward, there is both a clear need and an emerging trend toward integrative, human‐focused research programs that combine realistic food‐based interventions, robust biomarkers, and rigorous trial designs in diverse populations, including those currently under‐represented in the evidence base.

## Funding

This study was supported by Health Commission of Sichuan Province Medical Science and Technology Program (24WSXT082), Sichuan Provincial Administration of Traditional Chinese Medicine (25MSZX440).

## Conflicts of Interest

The authors declare no conflicts of interest.

## Supporting information


**Figure S1:** Temporal prominence of keyword clusters illustrated by the mountain/peak plot.

## Data Availability

The data for this study have been incorporated into the article.
