# Supplementary figures and images for: Global Research Trends in Dietary Polyphenols for Preventing Non‐Communicable Chronic Diseases: A Bibliometric Study
Source: Food Sci Nutr. 2026 Mar 2;14(3):e71539. doi: 10.1002/fsn3.71539 (PMC12951362; doi:10.1002/fsn3.71539)

**Figure S1. Temporal prominence of keyword clusters illustrated by the mountain/peak plot.**

**
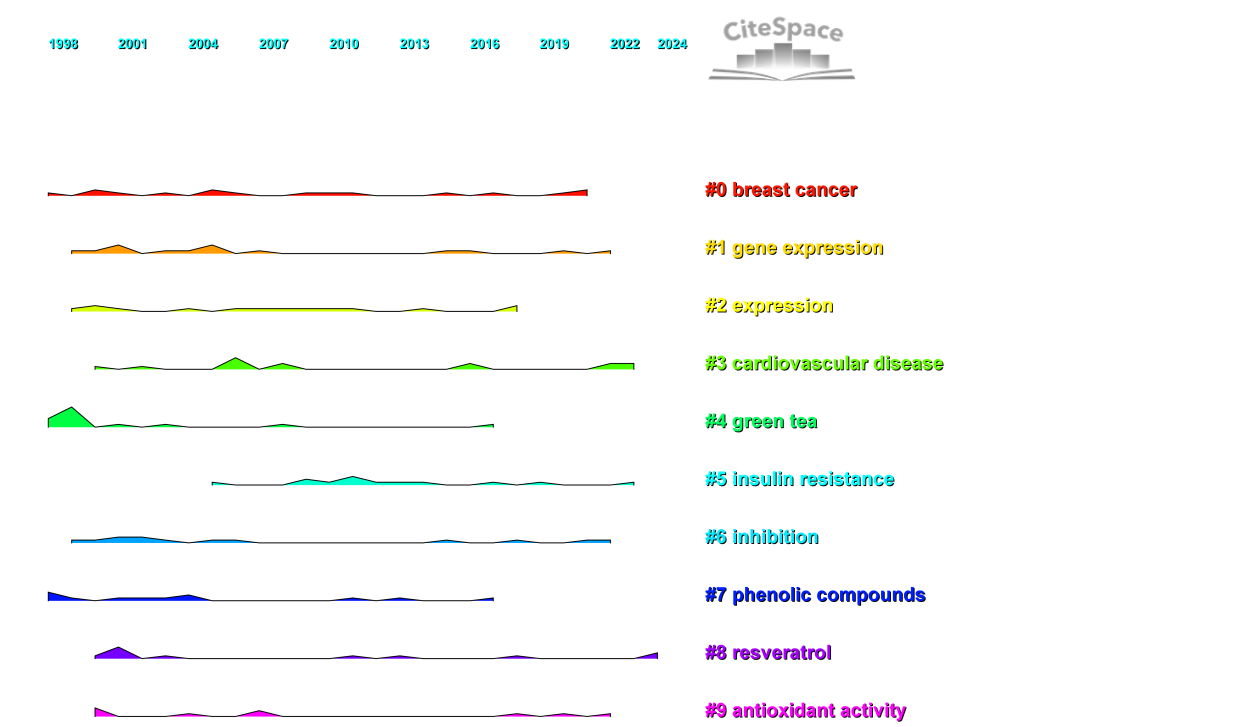
**

Supplement: Supplementary file 1 — Figure S1: Temporal prominence of keyword clusters illustrated by the mountain/peak plot. [file FSN3-14-e71539-s001.docx]
